# *Acinetobacter baumannii* Complex Infections: New Treatment Options in the Antibiotic Pipeline

**DOI:** 10.3390/microorganisms13020356

**Published:** 2025-02-07

**Authors:** Noayna Arshad, Wael Azzam, Marya D. Zilberberg, Andrew F. Shorr

**Affiliations:** 1 Department of Medicine, Medstar Washington Hospital Center, Washington, DC 20010, USA; noayna.arshad@medstar.net (N.A.); wael.azam@medstar.net (W.A.); 2 EviMed Research Group, LLC, Goshen, MA 01032, USA; evimedgroup@gmail.com; 3 Pulmonary and Critical Care Medicine, Medstar Washington Hospital Center, Washington, DC 20010, USA

**Keywords:** antibiotics, *Acinetobacter baumanni*, development, infection, pneumonia

## Abstract

Acinetobacter baumannii complex (ABC) can result in a panoply of severe syndromes, including pneumonia and septic shock. Options available for treating infections caused by ABC and, more importantly, by carbapenem-resistant ABC (CRAB) are limited because of the increasing prevalence of antimicrobial resistance. Furthermore, many older agents, such as polymyxin and colistin, have limited lung penetration and are associated with significant toxicities. These factors underscore the urgent need for new paradigms to address ABC and CRAB. Two agents, cefiderocol and sulbactam-durlobactam, are now available to treat CRAB infections. In addition, several anti-infectives that target CRAB are in later-stage clinical trials. In order to place these newer molecules in context and to help clinicians appreciate the emerging potential drug development pipeline, we describe the in vitro activity, mechanisms of action, and clinical trial data not only for the commercially now available alternatives, such as cefiderocol and sulbactam-durlobactam, but also review these topics for molecules undergoing phase II and III clinical trials. Specifically, we discuss and analyze data related to four novel drugs from ABC: BV-100, cefepime-zidebactam, zosurabalpin, and OMN6.

## 1. Introduction

Severe infection remains a leading cause of morbidity and mortality. Whether community or nosocomial in onset, infectious syndromes, ranging from septic shock and pneumonia to urinary tract and wound infections, represent an ongoing concern both for clinicians and patients. The challenge of bacterial infections is now particularly acute in light of the continuing increase in and spread of antimicrobial resistance (AMR) [1,2]. In recent years, select pathogens have evolved from being simply classified as “multi-drug resistant” (MDR) to now being considered extensively drug-resistant or pan-resistant (PDR). As a consequence, recent ordering schemes sort bacteria into more clinically relevant groups, such as whether the infection is “difficult to treat” (DTR) [1,2].

Although AMR has expanded substantially among both *Pseudomonas aeruginosa* and *Enterobacterales* species, *Acinetobacter baumanii* complex (ABC) presents a unique and growing concern. For example, the Centers for Disease Control and Prevention (CDC) lists ABC as an urgent threat [3]. Similarly, the World Health Organization (WHO) categorizes ABC as a critical threat necessitating urgent antimicrobial development [4].

ABC leads to trepidation among public health agencies for a multitude of reasons. First, ABC has developed and can evolve multiple mechanisms for AMR [5]. ABC often expresses changes both in the cell membrane and in efflux pumps that affect permeability and, in turn, the ability of antibiotics to attack the bacteria. The target sites for several small molecule antimicrobials may also shift in a way that renders them ineffective. More significantly, ABC possess intrinsic B-lactamases and can also acquire extrinsic B-lactamases. Second, and relatedly, ABC typically thrives within biofilm [6]. As such, it causes syndromes such as ventilator-associated pneumonia (VAP) and catheter-associated bloodstream infection (CLABCSI), which are associated with significant mortality. ABCs also produce their own intrinsic biofilms that may protect them from what would otherwise appear to be in vitro effective therapies. As a corollary, ABCs encased in biofilm are minimally metabolically active, which limits the efficacy of agents targeting protein synthesis. The extracellular matrix itself comprising the biofilm directly protects the pathogen both from many antibiotics and from the host’s immune system [7,8]. Hence, the effective armamentarium of agents available to treat ABC infections is rapidly diminishing.

Epidemiologically, ABC infections occur across the globe, but their prevalence varies widely from nation to nation. The highest rates of infection occur in Eastern Europe, Asia, and South America. A recent meta-analysis of 24 prevalence studies from Europe, the Eastern Mediterranean, and Africa concluded that infections due to ABC occurred in 0.85–5.6 cases per 1000 hospitalized patients [9]. The rate of ABC infection climbs to 56.5 cases per 1000 patients among those treated in the intensive care unit (ICU). A similar report focusing on Southeast Asia noted an incidence of ABC hospital-acquired infections (HAIs) in ICUs to be a staggering 18–649 infections per 1000 patients [10]. Conversely, in the US, ABC is a much less frequent cause of infection. CDC data suggest that there were fewer than 20,000 cases of ABC infection in 2017 [11]. Reflecting these global differences, in certain parts of the world where ABC is most prevalent, it is often implicated as the leading cause of HAI, accounting for 30% of all infections. In the US, on the other hand, recent data indicate that ABC causes fewer than 5% of all cases of VAP [12]. Irrespective of this wide range in prevalence, the risk factors for ABC remain consistent: being in the ICU, having co-morbidities, and requiring prolonged hospitalization. ABC can also ravage the immunocompromised, given their underlying inability to combat this pathogen.

Despite ABC’s varying prevalence from nation to nation, resistance to commonly employed first-line treatments for ABC is increasing uniformly at an alarming pace. Most notably, carbapenems can no longer reliably be prescribed empirically when clinicians suspect ABC. Carbapenem-resistant ABC (CRAB) is noted in 50–80% of cases seen in Asia and Latin America [9,10,11]. In one study in the US, prior to the COVID-19 pandemic, carbapenem resistance was seen in up to 50% of ABC isolates, while an analysis by the CDC-sponsored Emerging Infections Program in nine geographically diverse US centers found that more than 70% of ABC isolates displayed carbapenem resistance [11]. And during the pandemic, hospitals experienced a further surge in MDR overall and CRAB in particular.

Colistin serves as another key tool to treat ABC, generally, and CRAB, specifically. Colistin has many limitations (discussed below) but may be the only option for CRAB therapy in certain regions. Colistin acts via its electrostatic effect on the negatively charged ABC membrane and its lipopolysaccharide (LPS) components. Colistin resistance emerges due to modifications in the bacteria’s LPS [7]. These changes to the cell wall are mediated, in part, by select *mcr* genes, which, unfortunately, can be horizontally acquired. Some mcr genes, on the other hand (e.g., 4.3) do not confer colistin resistance. First identified nearly a decade ago ABC isolates with colistin-confirming *mcr* genes have now been observed in over 70 countries [7]. Despite being most often recovered in environmental samples, mcr genes containing ABC are increasingly seen in clinical settings. Presently, mcr appears to occur most frequently in infections arising in China. Other important genetically mediated mechanisms of colistin resistance relate to pmrCAB genes. The pmrCAB genes are part of a bacterial two-component regulatory system that plays a significant role in antibiotic resistance, particularly against polymyxins. Thus, the future role for colistin-based rescue regimens faces major challenges.

Its rapidly evolving resistance coupled with its overall burden renders ABC an urgent threat, as recognized by global public health agencies. Thus, clinicians require a comprehensive understanding of recently approved novel agents for the treatment of ABC and CRAB. In addition, those caring for patients with ABC infections must appreciate the evolving data supporting the potential of novel agents in the later stages of development. Two antimicrobials recently became available for treating ABC and CRAB: cefiderocol and sulbactam-durlobactam (SD). In addition, and demonstrating the robustness of the antimicrobial pipeline, four molecules for ABC and CRAB therapy are either in phase II or phase III clinical trials. Specific agents in more advanced human clinical trials include: zosurabalpin, BV-100, WCK-4234, and OMN6. To appreciate both the promise and the limitations of these agents, it is crucial to comprehend their in vitro activity and mechanisms of action along with the clinical data supporting their clinical use and/or continued development.

## 2. Materials and Methods

We completed a qualitative, narrative review of recently approved agents for treating ABC and CRAB infections. We also reviewed recent clinical trials and searched various drug development databases along with reports in the literature and corporate press releases to find and describe molecules under development for ABC and CRAB. We restricted our evaluation of the not-yet-approved drugs to those compounds in more advanced clinical trials (i.e., Phases II and/or III). For each agent, we have summarized not only the current in vitro potency data but also information regarding the mechanism of action and other relevant findings from clinical trials. For the recently approved antibiotics, we also summarize the key clinical trials that led to regulatory approval along with current real-world experience with these alternatives.

## 3. Results: Recently Approved Agents

### 3.1. Cefiderocol

An advanced generation cephalosporin, cefiderocol (see Table 1), is a newer option for combating ABC infections. The drug has been approved for use by regulatory authorities in the US, Europe, and parts of Asia. Cefiderocol has several distinct and unique mechanisms of action (MOAs) and represents the first successful siderophore antibiotic. It exploits the active iron transport apparatus of Gram-negative bacteria to enter the periplasmic space and thus avoids several of a bacteria’s inherent mechanisms of resistance [13]. The drug also binds to free iron in the cell and precludes the bacteria from metabolizing a key nutrient. Furthermore, this agent targets penicillin-binding protein 3 which helps inhibit cell wall synthesis [14]. Adding to its intracellular activity, cefiderocol does not serve as a substrate for the efflux pumps often contained in the cell walls of Gram-negative bacteria [15].

Because of these various aspects of the agent’s MOA, cefiderocol retains in vitro activity against many carbapenem-resistant organisms such as ABC, *P. aeruginosa*, and *Enterobacterales* spp. Demonstrating cefiderocol’s high degree of activity against all ABC, Kimbrough et al. conducted an analysis of over 1600 ABC isolates from both the US and Europe [16]. Nearly 60% of organisms tested were resistant to carbapenems (e.g., CRAB). Only 27 ABC isolates (1.7%) were cefiderocol non-susceptible at a break point of >4 mg/L [16]. Similarly, 2.7% of CRAB were non-susceptible to cefiderocol [16]. More strikingly, cefiderocol was the most active agent in vitro against ABC isolates, generally, and CRAB, specifically. Other large surveillance efforts have reported similar findings [15]. These data are summarized in Table 2.

Bacteria may develop resistance to cefiderocol via one of several mechanisms [14]. For example, B lactamase may evolve and lead to cefiderocol inactivation. Similarly, porin channel mutations and the development of efflux pumps also may lead to resistance. When encountered, bacteria resistant to cefiderocol often possess multiple potential mechanisms of resistance acting together and in concert. Changes in the key target penicillin-binding protein (PBP-3) may also lead to cefiderocol losing its in vitro activity. Because of its unique mechanism of action, mutations affecting siderophore receptors can further lead to the emergence of resistance [15].

The clinical data surrounding cefiderocol is more difficult to interpret. Two major randomized clinical trials were conducted during cefiderocol’s development that included patients with ABC infections. The first, APEKS-NP, a multicenter international trial, randomized 300 patients with Gram-negative nosocomial pneumonia, including VAP, to treatment with either high-dose meropenem or cefiderocol [17]. Overall, the clinical cure and mortality rates were similar between the two study arms. In the subgroup of patients infected with ABC (*n* = 47, 16% of the overall population), outcomes did not differ based on treatment regimen [17].

The second trial, CREDIBLE, was more complex. An open label, randomized study of best-available therapy vs. cefiderocol was restricted to subjects thought to be infected with carbapenem-resistant Gram-negative organisms [18]. This trial included a range of infections caused by different species of resistant bacteria. The final microbiologic intent-to-treat cohort included 118 patients, of which 80 received cefiderocol [18]. The main comparator in the remaining 38 study subjects was colistin, either as monotherapy or as part of a combination regimen. Nearly half of the infections (*n* = 54) arose due to CRAB. Despite its in vitro potency, the mortality rate in the subgroup of CRAB patients was statistically higher in the cefiderocol arm (49% vs. 18%, *p* = 0.04) [18]. Given the study’s sample size and design, there were substantial imbalances in the baseline characteristics of the two treatment groups, those in the cefiderocol arm being more severely ill. Although frank emergence of resistance on therapy did not occur often (given the formal breakpoints for cefiderocol), investigators noted that, in some patients treated with cefiderocol, the minimum inhibitory concentration (MIC) against cefiderocol increased several-fold. There seemed to be a correlation between this phenomenon and worse outcomes. Alternatively, the difference in mortality may reflect some issues with lung penetration and dosing viz. the MICs of CRAB relative to cefiderocol. In other words, for CRAB isolates with higher MICs against cefiderocol (yet still in the susceptible range) in patients with pneumonia, cefiderocol may not reach the needed targets to sterilize and control the infection. This potential explanation is only conjecture and may explain the findings of CREDIBLE. It does not align, however, with earlier animal studies [15]. The authors of CREDIBLE suggest a different explanation. They hypothesize that the issue is not the high rate of death amongst the patients with CRAB infections given cefiderocol, but rather the relatively and historically low mortality rate of subjects randomized to the best available therapy [18].

These confusing data initially made establishing the role of cefiderocol somewhat challenging. Newer evidence from both retrospective and observational analyses has helped clarify the potential role for cefiderocol in treating CRAB infections. Since its approval, multiple small case series and single-center analyses have been published and summarized elsewhere [19,20]. Most of these reports, because of their limited size and lack of a comparator arm, add little to the conversation about cefiderocol. More recent cases series, fortunately, have been larger and thus provide more insight. Piccica and colleagues described outcomes across three hospitals in 142 patients given cefiderocol, of whom 89 suffered from a CRAB infection [21]. The mortality rate was high (37%) in the CRAB-infected group, but the population was severely ill, with most patients requiring care in the ICU. Cefiderocol was well tolerated, and half of patients received cefiderocol monotherapy. Importantly, in both univariate and multivariate analyses, the monotherapy performed as well as combination cefiderocol treatment in terms of clinical success and mortality.

In a similarly sized multicenter Italian study of cefiderocol utilization, Giacobbe et al. reported outcomes among 65 subjects with CRAB infections given cefiderocol as either empiric or targeted therapy [22]. Confirming earlier observations, there was no difference in cure or mortality rates whether cefiderocol was given as monotherapy or as part of a combination therapy. More than a quarter of patients were treated empirically prior to a culture demonstrating CRAB.

An interim report of an international, observational, and uncontrolled study exploring cefiderocol utilization presented data on a subset of 244 individuals from the US treated with cefiderocol. Sixty-two of these subjects had either mono- or polymicrobial infections due to CRAB [23]. Although the median duration of cefiderocol use was 12 days, nearly half of patients were given more than 14 days of therapy. Clinical cure rates with cefiderocol remained high at approximately 75%, while the 30-day mortality rate was somewhat low (21%) relative to the historical experience with severe CRAB infections in seriously ill patients [23]. Consistent with the reports noted above, cefiderocol was well tolerated.

The findings from these larger case series imply that cefiderocol administration in the setting of CRAB infections results in generally good outcomes. However, the lack of a comparator arm makes it difficult to place such conclusions in the appropriate context. The results from analyses with a control arm, hence, are of more clinical value. For example, in a relatively large investigation (*n* = 124), Falcone et al. contrasted 47 cefiderocol treated patients to 77 individuals given a colistin-based regimen [24]. In the majority of cases, physicians concomitantly employed tigecycline. In an effort to create a measure of pseudorandomization, the authors relied upon an inverse probability of treatment weighting paradigm. In their adjusted analysis, cefiderocol treatment remained associated with a significantly lower risk of death [24]. Importantly, in the key subgroups of those with monomicrobial CRAB infections and persons with CRAB bacteremia, colistin use was also associated with higher mortality rates. However, this was not the case in the subgroup with CRAB pneumonia. The pneumonia group, though, was small (*n* = 35), and therefore, this observation must be interpreted with caution.

Addressing this specific question of cefiderocol in severe pneumonia, a group of Italian researchers assessed mortality in 122 individuals with VAP from CRAB who received either cefiderocol or alternative regimens [25]. Making this study distinct is the fact that all patients had COVID-19 infection and that many were treated with three anti-CRAB agents (as opposed to either mono- or two-drug combination therapy). Furthermore, nearly one-quarter of the population received both cefiderocol and colistin [25]. Unadjusted mortality was lower in those given a cefiderocol containing regimen (44% vs. 67%, *p* = 0.011). In a propensity score-based analysis designed to adjust for baseline imbalances in the two study arms, cefiderocol utilization continued to result in a greater probability of survival.

In an effort to summarize the entire published experience with cefiderocol for CRAB, Gatti and co-workers conducted a meta-analysis of all randomized clinical trials and retrospective comparative cohort studies reporting on cefiderocol [26]. Among 571 patients across multiple reports, in-hospital mortality was similar for those treated with cefiderocol vs. other regimens (mainly colistin) [26]. When examining only observational studies that adjusted for confounders, cefiderocol utilization was associated with a significantly lower risk for death.

In short, it appears cefiderocol is an appropriate option for some CRAB infections. Whether it results in improved survival as compared to treatment with known nephrotoxic agents with poor lung penetration such as colistin remains to be determined. At this point, guidelines from the Infectious Diseases Society of America (IDSA) do not list cefiderocol as a preferred agent for CRAB [27]. The guidelines indicate that cefiderocol, if used, should be reserved for patients failing other regimens, and should be employed as part of combination regimen and not as monotherapy. The European Society of Clinical Microbiology and Infectious Diseases (ESCMID) guidelines similarly recommend against the use of cefiderocol unless other agents are not appropriate [28]. In both guidelines, the main concerns about the use of cefiderocol revolve around difficulties with susceptibility testing in clinical practice, the potentially greater clinical utility of sulbactam in CRAB infections (thought no head-to-head comparisons exist), and apprehension about the CREDIBLE study along with the limitations of recent observational analyses.

### 3.2. Sulbactam-Durlobactam

SD (see Table 1) represents a combination of an older beta-lactamase inhibitor (BLI), sulbactam, with a novel BLI, durlobactam. Recently approved for use in the US, SD is undergoing regulatory review for potential approval in various Asian countries. However, because of the way the drug is currently manufactured and packaged, the drug does not comport with several current European Union regulations for pharmaceutical approval. Hence, until these issues are corrected, it remains unclear when this agent will become available for use in most of Europe.

Sulbactam has inherent in vitro activity against ABC because of its ability to bind to various penicillin-binding proteins often found in ABC isolates. Thus, clinicians have historically employed sulbactam as part of combination regimens for treating severe ABC and CRAB infections. Durlobactam, in contrast to sulbactam, is a novel diazabicyclooctane inhibitor that works against multiple Ambler Class D beta-lactamases and possesses direct activity against ABC organisms [29]. Interestingly, durlobactam helps to restore the activity of sulbactam against multiple enzymatic resistance mechanisms found in ABC and CRAB.

Two large microbiologic surveillance programs have described the in vitro potency of SD. First, Seifert and co-workers reported on the activity of SD in 246 CRAB isolates from over 30 countries [30]. The authors observed that, based on broth microdilution, the MIC90 for SD was 4 mg/dL for each agent individually. The MIC90 for colistin was similar at 1 mg/dL [30]. Of the 10 isolates that displayed several distinct mechanisms of resistance to colistin, all demonstrated low MICs against SD, and its in vitro activity did not vary across these various mechanisms.

Second, in a larger analysis of over 5000 ABC isolates, Karlowsky et al. described similar findings [31]. Among all ABC isolates (not just CRAB), the SD MIC90 was 2 mg/dL [32]. Importantly, the MIC90 of sulbactam alone fell substantially in the presence of durlobactam. Approximately half of isolates tested were resistant to carbapenems (i.e., CRAB). In these CRAB organisms, the SD MIC90 increased only by one dilution to 4 mg/dL. In the 4% of isolates that were colistin the resistant, the SD MIC90 remained at the 4 mg/dL level [29]. Unfortunately, neither of these investigations directly compared the in vitro activity of SD to cefiderocol. A summary of the in vitro studies is shown in Table 2.

Readers should note that the dose of SD of 1 gm of sulbactam along with 1 gm of durlobactam given every six hours over an extended infusion (3 h) is based on multiple careful pharmacokinetic (PK) and pharmacodynamic (PD) studies [33]. These evaluations indicate that the percent time above the MIC (%T > MIC) serves as the most appropriate PK/PD metric for sulbactam while for durlobactam the appropriate PK/PD target is the percent time spent above a critical time (CT) at 0.75 mg/dL [33]. Utilizing these values and various population PK models that include different degrees of renal function led to the final dose selection. The dose was also determined by taking the lung penetration of each agent into account and was confirmed in various animal and human models [33].

In terms of in vitro resistance, SD remains susceptible to the traditional mechanisms of resistance development noted with BLIs. In other words, B lactamases, the development of efflux pumps, and changes in porin channels can all promote resistance to SD. In practice, the emergence of B lactamases appears most important in the development of resistance to SD amongst ABC and CRAB isolates. In a systematic review by Principe et al., 100% of metallo β-lactamase-producing ABC strains demonstrated resistance to SD [32].

Based on these in vitro and modeling studies, along with earlier phase II clinical trials, SD was compared to colistin in a pivotal phase III randomized controlled trial. In the multinational ATTACK study, patients were randomized to treatment with either SD or colistin [34]. ATTACK represents the first pathogen directed trial for Gram-negative organisms. The study also employed a novel rapid diagnostic to facilitate early identification and enrollment of persons with CRAB infections [34]. All patients in the trial concomitantly received imipenem-cilastatin. This was done so as to provide broader coverage of potentially co-infecting Gram-negative pathogens, against which SD might not be effective.

The final study included 181 subjects, of which 125 comprised the microbiologic intent-to-treat population, the primary analysis cohort. Most subjects were critically ill and nearly 70% suffered from pneumonia. The study met its primary endpoint and demonstrated non-inferiority against colistin with respect to all-cause mortality at day 28. The mortality rate in those randomized to SD was less than 20% as compared to 32% in the colistin arm. Although not statistically different, and despite the fact that one cannot conclude superiority from non-inferiority trials, this 12% difference in mortality approached statistical significance [34]. Not surprisingly, there was less nephrotoxicity with SD than with colistin. Two additional observations suggested other potential clinical benefits with SD. First, the survival curves diverged early and at day 14 there was a survival advantage for SD over colistin. Second, the clinical cure rate favored SD.

Despite the signals of a possible benefit of SD over colistin, these data must be interpreted with caution. For example, although there were differences in the incidence of nephrotoxicity, both groups had the same rates of renal replacement therapy, the main concern of clinicians and the major driver of poor outcomes and prolonged lengths of stay with colistin [34]. This is likely because much of the difference in nephrotoxicity arose due to changes in the frequency of “at risk” renal injury as classified by the RIFLE criteria. Under the RIFLE scheme, “at risk” change represents a mild increase in the serum creatine and, hence, lacks clinical consequences. In other words, colistin seemed as well tolerated as SD. Likewise, nearly half of the patients had polymicrobial infections with CRAB and another Gram-negative organism [35]. The mortality rate in those with polymicrobial infections treated with colistin was higher than the rate of death among pure CRAB infected subjects given colistin. When restricting the analysis to a more relevant and easier to interpret population of monomicrobial CRAB infections, the sample size falls markedly. Moreover, within this monomicrobial population, the majority of deaths were not felt to have occurred due to the ABC infection. Specifically, only 33% and 47% of deaths were classified as infection-related in the SD and colistin arms, respectively [35]. Given these facts, the 28-day infection-related death rate in those with a monomicrobial CRAB infection treated with SD equaled 6% as opposed to 16% with colistin (*p* = 0.17) [35]. This fact reveals that (1) most patients with CRAB infections die with them rather than from them, and that (2) there does not appear to be a mortality benefit with SD over colistin.

A non-randomized, open-label arm of the ATTACK trial included subjects with either colistin-restraint CRAB infections or those who failed treatment with colistin. Among the 28 enrolled patients, most suffered from bacteremia as opposed to pneumonia, in contrast to what was seen in the randomized portion of ATTACK [34]. The mortality rate in this population was similar to that noted with SD in the randomized cohort and measured 18%.

In terms of treatment guidelines, the IDSA recommendations described above indicate that “the preferred regimen is sulbactam-durlobactam in combination with a carbapenem” for CRAB infections. Other agents should only be employed if SD is not available [27]. The authors of this recommendation argue for this approach because SD represents a combination treatment, which aligns with historical practices for severe CRAB infections. The results of the ATTACK study add to the strength of this guidance. Another preferred feature is that SD is a CRAB-targeted agent, as opposed to other options that might need to be reserved for other DTR pathogens such as metallo-beta-lactamase-producing carbapenem-resistant *Enterobacterales* [27].

Since SD has only been commercially available for a little over 12 months, there are very few case reports and/or observational descriptions of the real-world utilization of this agent. Tiseo and colleagues treated a burn patient with VAP due to CRAB that was also resistant to both colistin and cefiderocol [36]. The MIC of the isolate to SD was 4 mg/dL and the patient was treated with high-dose SD because she was undergoing continuous renal replacement therapy (CRRT). The subject received 17 days of therapy and survived. VanNafta et al. described a similar case in which a 44-year-old burn patient developed VAP due to CRAB on hospital day 63 [37]. Despite treatment with cefiderocol and eravacycline, the patient progressed to septic shock and was subsequently given several other rescue regimens. Again, because the patient was undergoing CRRT, and in conjunction with meropenem and tigecycline, high-dose SD was given under an expanded access program. The patient underwent 23 days of treatment and was discharged alive. These preliminary descriptions of recuse therapy are encouraging, but much more experience will be required to determine the appropriate setting for SD use, and to clarify its role as either an empiric alternative in instances of suspected CRAB infections or for as targeted treatment when CRAB is confirmed.

### 3.3. Agents in Development

#### BV-100

First approved in 1992, rifabutin is a spiropiperidyl rifamycin analog for treatment of disseminated *Mycobacterium avium* complex infection in patients with advanced human immunodeficiency virus (HIV) disease. Rifamycins have generally displayed decreased activity against Gram-negative organisms. These antimicrobials have a limited capacity to cross the cell’s outer membrane which limits their utility against such pathogens. A series of thoughtful and careful experiments, though, have actually revealed the opposite in the case of ABC. In fact, it now appears that rifabutin displays significant in vitro activity against ABC [38]. For example, an assessment in a nutrient-limited media unmasked the hyperactivity of rifabutin against CRAB isolates [39] The need to better understand rifabutin in a nutrient-poor, iron depleted media likely arises from the fact that this molecule acts in ways similar to cefiderocol, in that it acts via a siderophore. Furthermore, readers should note that compared to other rifamycins, rifabutin (BV-100, see Table 1) represents the most potent and, simultaneously, the least toxic agent in this class. The MIC against ABC, for instance, in one recent analysis equaled 0.0156 μg/mL in a depleted medium [40]. Other in vitro information regarding BV-100 is summarized in Table 2.

Resistance to BV-100’s main component, rifabutin, can evolve in one or a combination of several ways. Efflux pumps along with enzymatic changes can lead to ABC and CRAB isolates becoming resistant to rifabutin and, in turn, possibly to BV-100. The key mechanism of possible resistance, however, appears to be related to changes in the *rpoB* gene. This gene encodes for changes in RNA polymerase and inhibits the elongation of RNA [41]. This mechanism of resistance not only affects the susceptibly of ABC and CRAB to rifabutin but also to all rifamycins.

Additionally, one particular challenge related to the treatment of ABC and CRAB infections revolves around the potential for antibiotic resistance to emerge while on treatment. As noted earlier, this may be a relevant concern with cefiderocol. For rifabutin, fortunately, it appears that the emergence of resistance is less of a concern. More specifically, in vivo testing in animal models has documented that the emergence of resistance among ABC when exposed to rifabutin is exceedingly rare. In one report, this occurred at a frequency of less than 1.7 × 10^−9^ and 8.3 × 10^−9^ in rich and limited-nutrient media, respectively. Interestingly, less resistance emerged when animals were co-treated with colistin [41]. As colistin and polymyxins are commercially available across the globe, these observations suggest a potential and important hypothesis that merits testing in humans to answer the pressing question of whether co-administration of rifabutin with polymyxin could further help prevent the emergence of resistance during treatment.

At the same time, the lack of an intravenous (IV) formulation for rifabutin for clinical use limits its utility. Oral medications for the critically ill are often unreliable and have undepictable bioavailability and pharmacokinetics. If a patient is on vasopressors or getting active resuscitation, relying on an oral agent for a severe infection is considered imprudent. Pharmacologists at BioVersys, though, have successfully produced an IV form of rifabutin (BV100), and it is currently in clinical development.

Phase I trials with BV100 have documented the safety and tolerability of the drug, which is not surprising since rifabutin has been in use for over three decades [42]. The company recently completed a Phase II trial of BV100. In this small study, patients were randomized to receive BV100 along with polymyxin or polymyxin alone, as described in information available at Clinicaltrials.gov (NCT05685615). Subjects had to suffer from severe CRAB infections in order to be enrolled and had to have either a nosocomial pneumonia or a bloodstream infection. Although the trial has enrolled its final patient, no preliminary data have yet been released. The concomitant use of polymyxin in this trial is intriguing, as noted above, because methodologically, it allows the sponsor to include subjects with limited treatment options in light of antimicrobial resistance, while simultaneously exploring the potential utility of combining rifabutin with polymyxin to prevent the emergence of resistance.

### 3.4. Cefepime-Zidebactam

Further utilizing the technology of paring novel BLIs with older traditional beta-lactams, researchers are exploring the use of cefepime, a fourth-generation cephalosporin, in combination with zidebactam, a novel BLI. Specifically, zidebactam is a non-β-lactam penicillin that binds to protein (PBP) 2. A promising candidate, the drug combination (WCK 5222) is in early and expanding stage clinical development for treating resistant Gram-negative infections. One aspect that makes WCK 5222 (see Table 1) unique is that unlike many BLI combinations, which, with the exception of durlobactam, enhance activity against highly resistant *Enterobacterales* species and MDR *P. aeruginosa* but not ABC, it possibly possesses activity against ABC and CRAB isolates. Hence, this molecule may prove to have more clinical utility than those that have come before it, though this will have to be shown in clinical development.

Zidebactam is a bicyclo-acyl hydrazide BLI that not only selectively binds to PBP2 in Gram-negative bacteria but also inhibits a wide range of β-lactamases, including metallo-β-lactamases (MBLs) and class D enzymes [43]. This BLI therefore helps to avoid hydrolysis by β-lactamases and enhances the activity of cefepime, a PBP3-targeting antibiotic. This “enhancer effect” is particularly on display against OXA-type β-lactamase-producing pathogens, such as ABC generally, and CRAB specifically. Although most of the older cephalosporins lack in vitro activity against ABC, paring selected cephalosporins (e.g., cefepime and ceftazidime) with this BLI leads to a fourfold increase in in vitro activity against ABC [44].

A recent analysis evaluated the in vivo efficacy of cefepime-zidebactam against ABC strains in neutropenic mouse models of both lung and thigh infections [45]. The investigators noted a significant decline in bacterial burden vs. control and monotherapy groups and highlighted the BLI combination’s substantial anti-bacterial activity against 13 genotypically diverse, OXA-23/24 expressing ABC isolates [45].

Microscopic analysis in similar studies revealed spheroplast formation at sub-MIC levels of zidebactam, with additional augmentation of this effect in presence of cefepime. Zidebactam alone, however, does not elicit a bactericidal effect and requires a higher concentration of cefepime (16 µg/mL) to reach complete eradication at 24 h [45].

The SENTRY Antimicrobial Surveillance Program, which collected isolates from 137 global medical centers, evaluated the MICs of cefepime-zidebactam against a wide range of Gram-negative organisms using broth microdilution. For ABC, relatively higher MIC50 and 90 values (16/32 mg/L) were observed (Table 2). More importantly, there was little geographic variability in the apparent potency of cefepime-zidebactam, suggesting this agent may prove useful wherever ABC and CRAB are encountered. In addition, these MIC thresholds imply that higher drug concentrations may be required at the site of infection for ABC infections [46]. It must be made clear that controversy does exist about the potential for this agent in CRAB infections. A recent study of CRAB isolates recovered from patients in New York concluded that only 34% displayed MICs of ≤8 mg/L [47]. Although developed with cefipeme, it appears that this novel BLI improves the potency of sulbactam in a way somewhat similar to how durlobactam, acts in combination with sulbactam. For example, a combination of sulbactam/zidebactam combination restored sulbactam susceptibility in 91% of a group of 46 geographically diverse CRAB isolates—including isolates that were resistant to sulbactam/avibactam combination [48].

Readers should note that as a BLI at its core, zidebactam when combined with cefepime may lose in vitro activity based on a variety of traditional mechanisms of resistance ranging from the evolution of efflux pumps to the presence of inactivating enzymes. In particular, Class D carbapenemases (OXA-type), which can hydrolyze and inactivate the beta-lactam component of the zidebactam combination may render the antibiotic ineffective [49]. Unfortunately, little is specifically known about the emergence of resistance to cefepime-zidebactam.

With respect to clinical development, Wockhardt pharmaceuticals, the creators of cefepime-zidebactam are conducting a number of early clinical trials to verify the efficacy and safety of this cephalosporin/BLI compound. The pharmacokinetics and pharmacodynamics of the combination are well understood as is the drug combination’s lung penetration, a key issue to explore prior to conducting trials in pneumonia.

A phase III, multicenter, randomized study is currently recruiting for complicated urinary tract infections and compares meropenem to this cephalosporin/BL combination (NCT04979806). This is a traditional non-inferiority study. The current regimen pairs 2 g of cefepime and 1 g of zidebactam administered every 8 h. Although likely to include a few patients with ABC or CRAB infections, this study will be key to determining the safety of the molecule along with its efficacy. In addition, cefepime-zidebactam has been given as a rescue therapy for over 30 patients with severe, life-threatening infections thus, far and many of these subjects have recovered [49].

### 3.5. Zosurabalpin

Zosurabalpin (see Table 1) is a novel agent that represent a first in class macrocyclic peptide against Gram-positive and Gram-negative pathogens. This specific molecule includes a tripeptide subunit and a diphenylsulfide tether. As a consequence, it appears to have promising activity against ABC. Initially modified from an earlier molecule, zosurabalpin demonstrates 4 to 64 times improved potency against ABC relative to its progenitors. Recent in vitro analyses reveal this compound to be active against ABC, generally, and CRAB, specifically. Reported MICs against various ABC isolates range from ≤0.06–0.5 mg/L [50].

Further experiments underscore that zosurabalpin lacks activity against other Gram-negative pathogens, whether they be fermenters or non-fermenters. The absence of an effect on wild-type, efflux-impaired, and porin-deficient *E. coli*, *Klebsiella pneumoniae*, and *P. aeruginosa* indicates that macrocyclic agents appear to act at a unique target that is clearly distinct. With respect to the mechanism of action, follow on studies show that zosurabalpin attacks and interferes with the transport of lipopolysaccharide (LPS). Specifically, zosurabalpin acts at the inner-membrane LptB2FGC complex. In turn, this molecule impairs cell wall integrity [51,52].

In an additional in vitro potency analysis in 129 clinical ABC isolates, the MIC90 of zosurabalpin equaled only 1 mg/L [53]. Similarly, in a study of 450 ABC isolates from over 30 nations, zosurabalpin further exhibited impressive in vitro potency [53]. Importantly, the activity of zosurabalpin was similar in various media types, underscoring that microbiologic testing does not require a nutrient-rich or poor special media as other anti-ABC agents require [53]. A summary of in vitro potency is shown in Table 2.

Extending the preclinical data, in vivo PK studies in mice indicated good plasma exposure with high clearance, a low volume of distribution, and moderate protein binding. Not surprising in light of these PK parameters, zosurabalpin demonstrates dose-dependent efficacy in neutropenic mouse pan-resistant ABC pneumonia and thigh infection models.

Further suggesting the potential clinical utility of zosurabalpin, the spontaneous mutation rate that might lead to zosurabalpin resistance seems relatively low. The spontaneous mutation frequency for this molecule seems to range from 10^−7^ to <10^−9^ at 4× to 16× MIC—which is similar to the spontaneous mutation rates observed with current standard-of-care antibiotics for ABC. One potential mechanism for resistance within ABC and CRAB can evolve through point mutations in the LptF and LptG proteins—components of the LptB2FGC complex [51,52].

Given the molecule’s target, resistance will likely be potentially driven via mutations in the bacterial protein complex which serves as the agents target (e.g., LptB2FGC). Alterations in either or both of the LptF and LptG aspects of the target can prevent the antibiotic from binding effectively, thus rendering it inactive [51,52].

In the clinical realm, Roche has completed a series of early phase 1 trials with the agent. Single ascending IV doses of from 10 mg to 2000 mg of zosurabalpin were found to be safe, well tolerated, and displayed a predictable PK profile in healthy participants. Concerningly, investigators noted a dose-dependent infusion reaction in 14% of the participants, but it was fully reversible and mild [53]. Combination studies searching for potential synergism between zosurabalpin and other currently approved agents for ABC and CRAB did not show any synergism [54].

Roche may have begun recruiting for a larger clinical study of zosurabalpin but no details are publicly available regarding this trial. It is not evident whether this will be a traditional phase III trial or some form of a combined phase II/III trial. As two doses of the drug have been more thoroughly studied in phase I reports, no information is presently in the public domain about which dose will be taken forward.

### 3.6. OMN6

All the agents discussed thus far represent traditional antibiotics in that they are small molecules designed to target ABC and CRAB. Given the many issues with AMR because of the use and abuse of antibiotics, there is a need for a novel approach to attacking this pathogen. Non-traditional anti-infective agents therefore appear intriguing as possible alternatives for treating these severe infections. OMN6 represents one such potential future option. OMN6 is not an antibiotic, but rather, it is an antimicrobial peptide. Many insects and other creatures secrete antimicrobial peptides, such as the cecropin OMN6, to protect against infection. Generally, cecropins are small, positively charged compounds that, because of their structure, can bind to and penetrate cell membranes [46]. This in turn disrupts the membrane and results in bacterial death. Because of their construction combined with their mechanism of action, there is little potential for such antimicrobial peptides to promote antimicrobial or antibiotic resistance. Thus, traditional mechanisms of resistance may not be important concerns for OMN6. Nonetheless, theoretically, changes in the composition of the cell membrane of ABC and CRAB organisms could undermine OMN6’s in vitro activity [55,56]. In addition, genetic adaptations could lead to isolates acquiring gene-encoding to resist cecropins.

OMN6 (see Table 1) represents an artificial cecropin created to act on ABC. In vitro studies confirm its independent potency against ABC and CRAB strains. In one report exploring over 400 isolates of ABC and CRAB, OMN6 was active against 100% of the organisms tested [57]. Moreover, the MIC range was relatively low and narrow (4–8 mcg/mL) and OMN6 retained activity against CRAB strains, including those that were also colistin-resistant (Table 2). In the past, the development of other antibiotics has been hampered by the drug’s inability to penetrate lung surfactant or surfactant, inactivating the agent in development. Fortunately, the surfactant may, in fact, enhance the activity of OMN6, which makes it particularly intriguing for the treatment of ABC pneumonia [57].

When examined in mouse models of either MDR ABC bacteremia or pneumonia, OMN6 effectively treated both syndromes and resulted in substantial reductions in organism burden. In addition, these models reveal that the peptide can withstand proteolysis in vivo and also penetrate the lung effectively to sterilize the tissue [57].

Several clinical phase I studies have been completed. The drug has been well tolerated thus far with no significant adverse events. The PK appears predictable, dose-proportional, and not affected by patient age [58]. Omnix Medical, the developer of OMN6, has begun a phase II program with this unique antimicrobial peptide (NCT06087536). The trial aims to enroll persons with nosocomial pneumonia due to ABC and will study various doses of the drug on top of either meropenem or colistin. This design allows the inclusion of patients with either carbapenem-susceptible ABC or CRAB.

Most other anti-infectives under study for ABC are mainly focusing on CRAB given the high unmet need in this arena. OMN6, though, because of its unique design and mechanism of action, may prove beneficial for the treatment of all ABC infections, irrespective of whether the pathogen is carbapenem-susceptible or not. Similarly, if taken forward, OMN6 can be examined as both a stand-alone therapy as compared to traditional agents or as an adjunct to standard therapy. In this way, the use of a peptide is exciting, as it provides a way to examine the superiority of adjunctive therapy with different classes of molecules.

## 4. Conclusions

We now have two commercially approved agents for the treatment of severe infections due to ABC and CRAB. Each agent has unique attributes and distinct strengths and weaknesses (see Table 1). Clinicians are currently working to determine how best to employ these options so as to improve patient outcomes. Despite the availability of both cefiderocol and SD, though there remains a substantial unmet need in this space. The four agents in advanced development reviewed here may also be potential future options for ABC and CRAB. Their unique mechanisms of action, along with their potential to help limit the spread and development of further antibiotic resistance, holds the promise that we may soon have extremely effective clinical tools to combat challenging infections and reducing their mortality rates, which currently approach a staggering 40%. Reflecting the paucity of data about approved options and those in development, current guidelines make recommendations based on very limited data, and thus, clinicians continue to struggle with how best to utilize the current agents. Similarly, we have no studies directly comparing any agent against another in either and animal model or in humans for the treatment of actual infections. Such information is not likely to be available in the near (or even) distant future. As such, physicians will continue to struggle in their efforts to address ABC and CRAB infections. Hence, we will need to continue to strive through infection control and antibiotic stewardship to effectively contain ABC infections.

## Figures and Tables

**Table 1:** Summary of Recently Approved Agents and Those Under Development.

Agent	Manufacturer/Sponsor	Clinical Status	Mechanism of Action	Targets Only ABC (Yes/No)	May Be Adjunct in Susceptible ABC Infections (Yes/No)
Cefiderocol	Shionogi	Approved	Siderophore cephalosporin	No	No
Sulbactam-Durlobactam	Innoviva Specialty Therapeutics	Approved *	Combination agent with BLI with enhanced anti-ABC activity	Yes	No
BV-100 (IV rifabutin)	Bioversys	Phase II	Rifamycin	Yes	No
Cefepime-zidebactam	Wockhardt	Phase III	Novel BLI with anti-ABC activity	No	No
Zosurabalpin	Roche	Phase II/III	Novel macrocyclic peptide	Yes	No
OMN6	Omnix Medical	Phase II	Antimicrobial peptide	Yes	Yes

Abbreviations: ABC—*A. baumannii* complex, BLI—beta-lactamase inhibitor, IV—intravenous. * Only in the United States.

**Table 2:** In vitro Activity of Various Agents.

Agent	MIC Range (µg/mL)	MIC50 (µg/mL)	MIC90 (µg/mL)
Cefiderocol	0.001–4	0.12	1
Sulbactam-Durlobactam	0.016–64	1	4
BV-100	0.002–4	0.25	2
Cefepime-Zidebactam	0.002–32	0.5	4
Zosurabalpin	0.01–16	1	8
OMN6	0.004–32	0.5	4

Abbreviations: MIC—minimum inhibitory concentration. Data are summarized based on published studies discussed in the text.

## Data Availability

Not applicable.
